# Comment on: Community-acquired pneumonia: a US perspective on the guideline gap

**DOI:** 10.1093/jac/dkae201

**Published:** 2024-07-03

**Authors:** Carl Llor

**Affiliations:** Research Unit of General Practice, Department of Public Health, University of Southern Denmark, Odense, Denmark; University Institute in Primary Care Research Jordi Gol, Via Roma Health Centre, Barcelona, Spain; CIBER Enfermedades Infecciosas, Instituto de la Salud Carlos III, Madrid, Spain

A recent JAC article by Malinis *et al*.^1^ recommends using corticosteroids as adjuvant therapy for patients with community-acquired pneumonia (CAP). For patients with severe CAP, corticosteroids can decrease the risk of adult respiratory distress syndrome and modestly reduce ICU and hospital stays, the duration of intravenous antibiotic treatment, and the time to clinical stability, without increasing major adverse events.^2^ Additionally, a recent systematic review found that oral corticosteroids can reduce the risk of mortality in patients with severe CAP by 34% but have no effect in non-severe cases.^3^

Up to 80% of CAP treatment occurs in outpatient settings, with the remaining 20% requiring hospitalization.^4^ The clinical presentation of CAP ranges from mild to severe, with mortality rates varying widely based on treatment setting and disease severity, being <1% for most primary care and ambulatory patients.^5^ In outpatients with acute lower respiratory tract infections (LRTIs) who do not have chronic obstructive pulmonary disease, treatments such as cough suppressants, expectorants, mucolytics, antihistamines, inhaled corticosteroids, and bronchodilators have not proven to be effective in those without underlying lung disease.^6^ A systematic review found insufficient evidence to recommend inhaled corticosteroids.^7^ Additionally, a randomized clinical trial evaluating 40 mg of oral prednisolone for 5 days in adults without asthma found that corticosteroids did not reduce the duration of moderately bad or worse cough or symptom severity in patients with acute LRTIs.^8^ Neither is there any evidence to support corticosteroid use for LRTIs in patients with undiagnosed asthma, as the duration of cough was found to be the same in both the prednisolone and placebo groups.^9^ Nonetheless, no specific study has evaluated the role of oral corticosteroids in patients with CAP managed in the community.

Malinis *et al.*^1^ mention that ‘corticosteroid therapy is likely to become standard of care for patients with CAP’. However, based on the results of randomized clinical trials, this adjunctive therapy should be limited to patients with severe pneumonia, which constitutes a small percentage of patients with CAP. There is still insufficient evidence to recommend specific non-antibiotic, symptomatic therapies for outpatients with CAP. Large-scale randomized clinical trials are needed to investigate the role of oral corticosteroids in subpopulations other than severe CAP.
